# Masculinities in a feminist pedagogy: lessons for transformative gender and agriculture training

**DOI:** 10.3389/fsoc.2024.1461445

**Published:** 2024-11-06

**Authors:** Amon Ashaba Mwiine, Margaret Najjingo Mangheni, Elizabeth Asiimwe, Martha Businge, Fred Shimali, Losira Nasirumbi Sanya

**Affiliations:** ^1^School of Women and Gender Studies, Makerere University, Kampala, Uganda; ^2^Department of Extension and Innovation Studies, Makerere University, Kampala, Uganda; ^3^Gender-Responsive Researches Equipped for Agricultural Transformation (GREAT) Project, College of Agricultural and Environmental Sciences, Makerere University, Kampala, Uganda

**Keywords:** masculinities, agriculture, gender training, gender analysis, gender transformation

## Abstract

Masculinities and femininities are closely interconnected with men and women farmers’ everyday lives; hence critical reflection on these interconnections should be central in gender training in agriculture. While a focus on men and masculinities is crucial for sustainable transformation of deep-rooted gender norms and practices that limit the attainment of gender equality, there are insufficient empirically tested pedagogical models for this purpose. We share a case study, the Gender Responsive Researchers Equipped for Agricultural Transformation (GREAT) model, which incorporates masculinities in a feminist pedagogy. We use external monitoring, evaluation, and learning data for two case study courses that integrate gender in plant breeding, seed systems, and agronomy to demonstrate the efficacy of integrating the concept of masculinity and reflections on male farmers’ expectations, behaviors, and practices within a feminist approach to gender training. We conclude that feminist pedagogical practices offer insights into how gender training can integrate a masculinities perspective to move beyond divisive and narrow gender polarities towards addressing masculine norms that often hinder the attainment of gender transformation.

## 1 Introduction

There is a steady growth of scholarship on men and masculinities globally, including in agricultural research (Brandth, 1995; Sweetman, 1997; Saugeres, 2002; Cole et al., 2015; Bonatti et al., 2019; Hillenbrand and Miruka, 2019; FAO, IFAD, and WFP, 2020). This paper reflects on the journey of integrating masculinity perspective in the Gender Responsive Researchers Equipped for Agricultural Transformation (GREAT) training courses that are heavily informed by feminist pedagogy. On the one hand, masculinity is defined as “the set of social practices and cultural representations associated with being a man” (Pilcher and Whelehan, 2004, p. 82). The plural “masculinities” is often used in recognition that ways of being a man and cultural representations of/about men vary, both historically and culturally, between societies and between different groupings of men within any one society. Initially conceptualized in sociological studies, the focus on men and masculinities has expanded to different fields of knowledge including feminist studies, agriculture, and technology. On the other hand, feminist pedagogy refers to a theory of teaching and learning that emphasizes the learning space as a “liberatory environment” (Shrewsbury, 1987). Caroly Shrewsbury (pp. 6–7) particularly notes that “feminist pedagogy is concerned with gender justice and overcoming oppressions” Feminist pedagogy privileges critical and reflective learning by reframing the relationship between teacher and learners, empowering the learners by building a community, privileging their voice, respecting diverse personal experiences, and challenging traditional views.

In the early 1990s, Brandth (1995) and Saugeres (2002) contributed to earlier intersectional debates on masculinities in agriculture, especially looking at what it means to be masculine in this field. Saugeres examined how cultural constructions of masculinity in a farming community in southern France were articulated around ideas of masculine power symbolized by agricultural machinery –notably tractors. Around the same time, the focus on men and their sense of self as ‘masculine’ and the relevance of this for development was gaining ground through Gender and Development (GAD) approaches (Sweetman, 1997; Cornwall, 2000; Cleaver, 2002). Sweetman (1997, p. 2) argued that “men and masculinity need to be studied if power relations between the sexes are to be changed for the better, and the potential of individuals of both sexes to be realized.”

Gender and Development discourse was and continues to be preoccupied with the promise of a new focus beyond the narrow concern of Women in Development (WID) with women alone. WID came into existence as an approach that sought to tackle women’s subordination through an explicit emphasis on socially and historically constructed relations between women and men (Cornwall, 2000). This conceptual shift is re-echoed by Connell (2005), who observed that while women pioneered the gender equality agenda, mainly because of the pervasive oppression they experienced in patriarchal systems, the idea that men might have a specific role in relation to this principle only emerged later. It was argued that moving toward a gender-equal society involves profound institutional change as well as change in everyday life and personal conduct. “To move far in this direction requires widespread social support, including significant support from men and boys” (Connell, 2005, p. 1801). Consequently, the global shift from the terminology of WID to GAD was indicative of efforts towards bringing men and masculinities into the picture.

### 1.1 Shift towards masculinities in gender and agriculture research

Recent shifts in agriculture (and indeed other fields of knowledge) indicate a renewed focus on examining how norms around being a man, questioning negative masculinities, and working with boys and men to nurture positive masculinities is prerequisite to gender transformation (Hillenbrand and Miruka, 2019; FAO, IFAD, and WFP, 2020). Debates on masculinities in agriculture occur in the broad context of interactions between gender theories, agriculture, and development practice. Beyond the masculine nature of agricultural technologies (Brandth, 1995; Saugeres, 2002), the concept of masculinities has also been discussed in relation to women’s empowerment (Bonatti et al., 2019; Santoso et al., 2019; Ambler et al., 2021), poverty and rural agricultural livelihoods (Sraboni et al., 2014; Cole et al., 2015), social and gender norms in agriculture (Hillenbrand and Miruka, 2019), nutrition and food security, and most recently, gender transformative approaches in agricultural research (FAO, IFAD, and WFP, 2020).

Studies on women’s empowerment in agriculture such as Bonatti et al. (2019), on masculinities and femininities in food insecurity in Tanzania, show women’s role in the food production process as food producers, family food managers, and consumers. They argue that even when women contribute significantly to food availability, men continue to play a dominant role in the decision-making process. Equally, Ambler et al. (2021) and Lecoutere and Wuyts (2021) reflect on innovative approaches of involving men and women as couples in intra-household decision-making to facilitate women’s economic empowerment. In their work exploring the intricate relationship between poverty, gender inequality, and rural masculinity in aquatic agricultural systems in Zambia, Cole et al. (2015, p. 155) highlight the need to focus on masculinities to address persistent gender inequalities so as to transform gender power relations. They critique polarization of women and men by arguing that “research for development initiatives that focus on the ‘separate characteristics of women and men rather than on the way that social institutions work together to create and maintain advantages and disadvantages’ are highly problematic and fail to sustainably reduce gaps in poverty between women and men.” This critique is followed by an elaborate use of the concept “masculine-rural” to explain how men’s behaviors, practices, beliefs or norms are socially constituted in rural areas and the implications these have for women’s lived experiences. These studies thus called for an understanding of femininities and masculinities, along with their influence on and interconnections with food systems, because these gendered relations are not only situational but also intricately too linked to be dealt with in isolation.

Further, efforts to understand masculinities have equally focused on gender norms in agriculture and how to transform these to ensure equitably sustainable agricultural systems (Hillenbrand and Miruka, 2019; FAO, IFAD, and WFP, 2020). Hillenbrand and Miruka (2019) highlight increasing global recognition of the diversity of masculinities and how norms and performances of masculinity vary culturally and contextually, with expectations differing by class, race, and age. FAO, IFAD, and WFP (2020) highlight explicit engagement with men and boys to address the concepts of masculinity and gender as a critical step towards challenging deep-rooted social and gender norms. In some cases, agricultural research has adopted specific methodologies to engage men and boys, such as Journeys of Transformation or Engaging Men as Allies in Women’s Economic Empowerment. It is argued that through such methodological innovations that facilitate “personal reflection and dialogue, men begin to see how rigid constructions of masculinity not only can lead to harm for their partners but also for themselves, and see the benefits of more equitable relationships” (FAO, IFAD, and WFP, 2020, p. 65).

### 1.2 The problem

Despite shifts in theoretical and development approaches and their emphasis on gender analysis that includes addressing masculinities as part of gender relations, the focus on men and masculinities in gender training and research programmes remains minimal and, in some cases, nonexistent. There remains limited focus on the concept and theories of masculinities in agriculture training programmes. Agricultural research encounters with masculinities remains limited to unpacking the concept of masculinity and what this means for different agricultural communities. In other cases, certain approaches to engaging men remain instrumentalist – involving men to benefit women agricultural farmers rather than targeting transforming relations of inequality between women and men. This paper seeks to address this gap tracing the journey of GREAT (Gender Responsive Researchers Equipped for Agricultural Transformation) training courses. GREAT is a joint, multi-disciplinary, short gender training course for agricultural researchers designed and delivered by Makerere University in Uganda and Cornell University in the US. The model introduced sessions on men and masculinities in its training curriculum in the context of feminist pedagogy principles.

The paper thus highlights ways in which the concept and debates on men and masculinities in agriculture were integrated into a feminist-oriented pedagogy, the participants’ perception of this innovation, and implications for future trainings in gender and agriculture.

### 1.3 Brief insights about the GREAT training model

The GREAT model, initially designed in 2015 for Sub-Saharan Africa, was motivated by the need to have a comprehensive, trainee-centered, critical, interdisciplinary, and practical gender training course that would provide space for reflection on internalized gender beliefs, biases, and identities and foster gender transformation (Mangheni et al., 2019). The GREAT course “pioneered a training model that seeks to challenge the status quo of crop improvement and agricultural research while confronting entrenched gender norms and triggering attitudinal shifts and practice change” (Mangheni et al., 2021, p. 42). The course adopts an in-depth phased training model for tailored skills development in gender-responsiveness along agricultural research design, implementation, evaluation, and communication. GREAT trains interdisciplinary teams of biophysical scientists (plant breeders, agronomists, entomologists, and pathologists) and social scientists (anthropologists, sociologists, economists, and gender specialists) together. The overall learning objective is “to strengthen the ability of agricultural researchers to design, conduct and communicate gender-responsive research.” Topics covered range from gender concepts, personal reflections on gender relations, gender biases, and positionality to applied sessions on gender-responsive social research methods and gender-responsive plant breeding and seed systems (Mangheni et al., 2021). See Mangheni and Tufan (2022) for the detailed GREAT trainers’ manual.

Since the first GREAT course in 2016, the training has reached 346 agricultural researchers, from 33 countries and 75 institutions. Notably, 43% of the participants that have gone through the trainings are women, and 57% are men (GREAT, 2023).

It is within this context of commitment to foster gender transformative change in agricultural research that GREAT introduced the focus on men and masculinities in its training sessions. The goal was to provide an opportunity for agricultural researchers to move beyond gender approaches that focused on women farmers in exclusion towards understanding the role of men’s practices and expectations in framing agricultural outcomes in the household, communities, and institutional policy level.

## 2 Methodology and theoretical approach

### 2.1 Research design and case study description

We used a case study design employing multiple cases, data sources, and data collection methods to enable an in-depth understanding of the study phenomena. The choice of design is informed by Simons (2009, 1), who defines a case study as “an in-depth exploration from multiple perspectives of the complexity and uniqueness of a particular project, policy, institution, program, or system in a real-life context.” In addition, Stake (1995) indicates that case study research is concerned with complexity, while Creswell (2002, p. 61) defines it as a problem to be studied, which will reveal an in-depth understanding of a “case” or bounded system, which involves understanding an event, activity, process, or one or more individuals. The multi-disciplinary GREAT courses which attracted participants from diverse cultures, disciplines, and organizations; were offered by trainers from equally diverse backgrounds; and commissioned by a range of organizations, qualify for multi-perspective in-depth analysis afforded by the case study design.

At the time of the study, GREAT had conducted a total of 15 courses targeting the agricultural research themes of plant breeding and agronomy. The study focused on two training courses as case studies, representing the two thematic areas of plant breeding (Case 1) and agronomy (Case 2). We selected the cases where course monitoring learning and evaluation (MLE) data revealed the most profound testimonies of significant personal discovery and attitude change amongst participants after attending the masculinities sessions. The two purposively selected cases are best suited to demonstrate the pedagogical lessons of what works.

The Gender-responsive plant breeding and seed systems: application to South Asia course (Case 1) participants were competitively selected after an open application call that attracted applicants from 12 institutions in three South Asian countries (India, Bangladesh, and Nepal). Case study 2, “Delivering gender-and youth-responsive agronomic solutions,” was commissioned by the Excellence in Agronomy (EiA) Initiative of the One CGIAR and targeted research teams funded by the initiative. The table below profiles both case studies (see Tables 1, 2).

**Table 1:** GREAT courses selected for this study.

Course features	Case 1: gender-responsive plant breeding and seed systems: application to South Asia Course	Case 2: delivering gender- and youth-responsive agronomic solutions Course
Training approach	2-parts (part 1 for both biophysical and social scientists; part 2 for social scientists, and optional for biophysical scientists)	One part targeting all participants
Course duration (dates)	11 days (part 1: 12-17th; part 2: 26-30th September 2022)	Five days (27th Feb- 3rd March, 2023)
Delivery approach	Blended (6 days face-to-face; 5 days virtually synchronous)	Face-to-face
Location (Region, city, country)	South East Asia (Hyderabad, India)	Sub-Saharan Africa (Kigali, Rwanda)
Number of participants	Part 1: 27 (8 women, 19 men); Part 2: 9 (5 women, 4 men)	23 (8 women, 15 men)
Participants’ countries of origin	India, Nepal, Bangladesh	Kenya, Ghana, Nigeria, Benin, Ethiopia, India, Cambodia, Malawi, and Mexico
Participants disciplines	Biophysical and social scientists	Biophysical and social scientists
Focus of participants’ work	Plant breeding and seed systems research programs	Agronomy-related projects
Focus of course content	Concepts and principles of gender-responsive plant breeding and seed systems; Entry points for gender integration in crop breeding and seed systems; and How to collect, analyze, interpret, and integrate gender data that accounts for diverse experiences and social identities into plant breeding and seed systems.	Gender concepts and why gender and youth inclusion matters in agronomy innovations; Frameworks for gender and youth integration in the EiA workflow; Gender and social analysis; The EiA Standard Operating Procedures (SOPs) for gender and youth integration; Engaging women, youth and, Gender-responsive scaling

**Table 2:** Participants’ rating of the sessions related to masculinities.

Session	Participants’ mean rating of the sessions	
	Case 1	Case 2
Gender and why it matters in agriculture; concepts on gender and social difference	3.59	3.83
Personal reflections on gender	3.48	3.74
Introduction to masculinities: implications for plant breeding and seed systems	3.56	3.70
Gender transformative approaches	3.33	3.78

Source: MLE reports.

The courses occurred in social-culturally diverse contexts of gender, class, ethnicity, caste, religion, age, geographical location, covering different themes in agricultural research. These contexts enabled understanding of the complex gender relations including different forms of masculinities.

### 2.2 Data collection methods

The researchers were immersed in the case study implementation processes. All authors are African feminists working and staying in Uganda, East Africa. The first author (a man) was the lead trainer of the independent session on masculinities and agriculture; the second author (a woman) was a trainer and principal investigator in charge of overall course design and oversight on implementation. The third author (a woman) was in charge of course coordination and management, and the fourth and fifth authors (woman and man, respectively) were course participants in previous GREAT courses. The sixth author (a woman) was a participant in the GREAT course in 2016 and was later recruited as a trainer. The authors were therefore sources of data having been interviewed by the external MLE team; later stepping back to synthesize and reflect on the findings to draw out meaning and conclusions presented in the paper.

The study used data from GREAT’s robust Monitoring, Learning, and Evaluation (MLE) dataset. The MLE system was designed by an independent agency separate from the trainer team to provide both real-time and long-term objective insights on the process and outcomes of the course to a range of stakeholders. Understanding the value of the different components of the training, delivery methods, and the extent to which participants’ needs and expectations were met was critical to continuously improving the course. The MLE system collected data on how novel features of the GREAT training, including efforts to challenge existing biases and positionality, were perceived and valued by participants alongside an appraisal of training content, tools, and delivery. The MLE partner developed the overall MLE system, tools and data collection in consultation with the GREAT program team who are co-authors of this paper. Travis et al. (2021) present a detailed description of the GREAT MLE methods which included participant observations; document reviews; participant surveys; trainer debrief meetings, and annual reflection sessions of trainers and program team. The paper analyzed relevant MLE reports, primary qualitative data from trainers’ systematic reflections, and post-course participant surveys and key informant interviews to understand participants’ reactions and feedback on the value of masculinities in gender-responsive agricultural research and their experiences with the training sessions.

#### 2.2.1 Trainer and course management team reflections

Debriefs by trainers and course managers occurred regularly. During the courses, the entire training team met at the end of each day for discussions (1–2 h) of the day’s sessions. Trainers used sticky notes to track the pros, cons, and changes in each session, with a brief review of all comments. Trainers reflected and discussed each session and general aspects of the model based on the compiled comments and suggestions from the daily briefings during the course. In addition, the team reflected on the entire course at the end and annually on courses held during the year. The reflections were systematically documented and synthesized into an overall report.

#### 2.2.2 Post-course participant survey

Data were collected electronically through a self-administered post-course survey (Case 1 *n* = 27, Case 2 *n* = 23) administered through a Google form sent out immediately after the end of the course. With both closed and open-ended questions, the post-course evaluation sought to assess participant satisfaction with aspects of the training such as delivery approach, course content, sessions, logistics as well as the duration of the course. The survey evaluation used a four-level scale to assess participant satisfaction with the various aspects where: 4 = extremely satisfied, 3 = satisfied, 2 = partly satisfied, or 1 = not satisfied at all; and a five-level rating scale to assess their proficiency in key competencies targeted by the course, where: 5 = Very high proficiency and 1 = very low proficiency. Open-ended questions allowed respondents to include more information for the project team to better understand the respondents’ feelings and attitudes about the course (ALINe Impact, 2022; GREAT and IITA, 2023).

#### 2.2.3 Key informant interviews

Key informant interviews were conducted with 4 course participants carefully selected to balance representation by region, gender, and discipline. These provided open ended qualitative insights and perspectives on the course to complement the surveys and trainer/project team reflections. Interviews were held virtually over the Zoom platform for 30 min to 1 h. Questions revolved around whether participant’s expectations were met; key learnings; perceptions on various aspects of the course training content, delivery approaches, trainer competency, logistics; general impressions of the course, and what could be improved. Interview data were transcribed verbatim and content analysis used to generate key themes. In MLE reports, themes were triangulated with the quantitative results to explain and interpret convergence/divergence in opinions.

### 2.3 Theoretical framework

This study was informed by two theoretical perspectives, i.e., Shrewsbury (1987)’s work on feminist pedagogy and RW Connell’s theory on hegemonic masculinity. In pedagogical theory, Shrewsbury (1987, pp 6-7) highlights feminist pedagogy as a teaching and learning process that emphasizes the classroom as a “liberatory environment,” collective knowledge production, democratic process in which at least some power is shared. Importantly, “feminist pedagogy is concerned with gender justice and overcoming oppressions” Feminist pedagogy privileges critical and reflective learning by reframing the relationship between teacher and learners; empowering the learners by building a community, privileging their voice, respecting diverse personal experiences, and challenging traditional views. On the other hand, Connell’s theory on gender order looks at hegemonic masculinity as a specific form of masculinity in a given historical and society-wide social setting that legitimates unequal gender relations between men and women, between masculinity and femininity, and among masculinities (Connell, 1995). As a dominant form of male identity, hegemonic masculinity is characterized by men’s authority, decision-making, household headship, controlling key resources, domination (class, race, caste, ethnicity) ambition, risk- taking, and ultimately, subordination of women and children. Men who fail or consciously make a decision not to fit in this model occupy other forms of masculinities such as ambivalent, subordinate, marginal masculinities. These theoretical insights reveal the social processes through which masculinities are constructed and the hierarchies, diversities, and relationality within which men’s experiences present in everyday life (Connell, 2005).

While Connell’s work conceptualises what can be termed as ‘breadwinning masculinity’, one of the resilient forms of masculinity, Su (2024) goes beyond this to theorise tensions within masculinities, especially amidst global changes and changing femininities. In their work on “Striving to be Men in the Family,” Su draws on examples of Vietnam transition from communism to capitalism to highlight men’s diverging views about women’s work. The paper traces the performance of masculinities amongst men with low-paid wage occupations and men employed in higher paying salary-earning occupations. The author contrasts these two economic contexts to indicate how men in low-paid jobs aspired for the notion of ‘traditional’ male breadwinner and a caretaker wife while the men in salary-earning contexts preferred a dual-income family, viewing of women workers as progressive. This theoretical perspective reinforces the idea of masculinity as socially constituted, relational (constituted in relation to femininities), and liable to change, especially amidst the transformation of socio-economic contexts. The idea of masculinity as complex, fluid, and relational creates a sense of optimism especially with regard to progressive forms of masculinities and bolsters our call for feminist pedagogies to deliberately engage with masculinities for meaningful transformation of unequal gender relations in agricultural communities.

The conceptual locus of this paper equally includes a focus on the theory of gender transformation that highlights a trajectory from gender blindness to gender aware and, consequently gender transformation. Engaging with the question of men and masculinities is at the center of gender transformative approaches, which seek ways to achieve profound and sustainable development objectives by tackling the root causes of gender inequality. As such, gender transformation does require a focus on gender equality and women’s empowerment alongside masculinities, since “challenging the traditional views of masculinity enables men to live positively, and work and live with women as equals” (FAO, IFAD, and WFP, 2020, p. 15).

The GREAT model is based on the premise that social constructions can be learned and unlearned through the active participation of affected parties to redefine the “ill situation” (Luckett and Luckett, 2009). The course was conceived as a feminist pedagogical model in as far as it emphasized critical examination of gender power dynamics, centering marginalized voices in agricultural practice, promoting inclusivity and equity and encouraging critical reflections and gender transformation through agriculture research. Integrating masculinities perspective was thus perceived as central to achieving the goal of gender transformation in agricultural communities.

## 3 Presentation of findings

The results presented in this section reflect *how* the concept and theoretical debates on men and masculinities were introduced in the feminist pedagogical trainings. The paper equally presents findings on how trainees *received* and *perceived* the training sessions on men and masculinities. Post-training assessments revealed that participants were generally satisfied with all course sessions (average scores above 3.5 out of a possible maximum of 4).

### 3.1 Integrating masculinities approach in the GREAT pedagogical model

**Figure 1:**
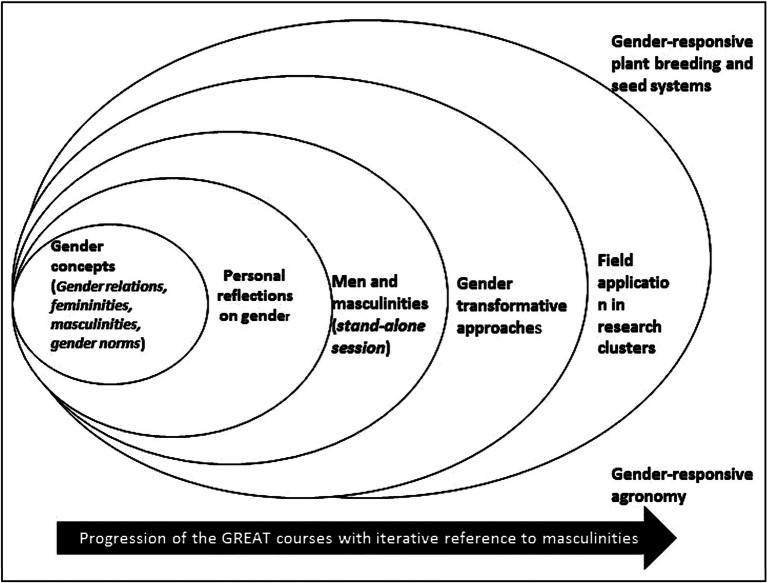
The positioning of masculinities in the GREAT case study courses 1 and 2.

The learning journey adopted a stepwise approach, intentionally weaving the masculinities message in progressive sessions (see Figure 1): first, was the session on definition of gender concepts; followed by one on personal reflections on gender to understand self and how gender manifests within workplaces and other social institutional spaces. This was followed by an independent session on men, masculinities, and agriculture; and finally, one on addressing masculinities and gender norms within the gender transformative approaches. The theoretical sessions were complemented with field research on gender and agriculture using a masculinities approach. This paper reflects on this pedagogical journey tracing learnings from each of the stages and the implications these approaches have for transformative gender trainings.

### 3.2 The focus on masculinities in the definition of gender concepts

GREAT training often begins with a session on gender concepts. Earlier trainings focused on concepts such as Gender, Sex, Inequality, Equality, Equity, Discrimination, and many others. With the introduction of masculinities perspective to these trainings, the first session introduces the concepts of gender, gender relations, defines masculinity alongside femininity, gender norms, patriarchy, and women empowerment. It includes examination of the experiences of men in agriculture communities to broaden participants’ understanding of gender relations.

Trainers use a series of participatory reflexive exercises to enable participants understand what gender means. Pictures drawn from specific cultures and day-to-day lives help to stimulate reflection and discussion on socially constructed roles, e.g., men as main breadwinners, women as homemakers, and caregivers; gender relations, e.g., men as leaders in homes, women as subordinates; and different men and women’s realities versus gender stereotypes. The pictures are used to trigger critical discussions on what is acceptable or not to help challenge what is taken for granted. Experiential reflexive pedagogical approaches facilitate progressive realization and internalization of how gender is learnt and reproduced explaining socialization and gender norms (Shrewsbury, 1987). Emphasis is that gender is defined as a socio-cultural system that organizes identity and relations between men and women in society (Ridgeway and Correll, 2004), and hence any discourse on gender should include men and women.

Trainers facilitate a reflection on the concepts of femininities and masculinities. Participants are asked to reflect on their cultural repertoire to define and explain the differences between masculinity and femininity. Their submissions, which are often culturally diverse, are used to explain the concepts bringing out the interrelatedness in definitions of femininity, masculinity, and gender norms. Within Case 1 discussion groups, participants appreciated the social construction of masculinities. They observed that while men hold preponderant power in agriculture systems, gender-sensitive agriculture programmes often miss out on examining men’s power, ultimately missing the opportunity to change dominant positions that men hold which deter agricultural development. Similar revelations were noted in Case 2 where participants from Eastern Africa, Southern Africa, South Asia, North Africa, and Latin America, engaged with the question of who a man is in an ideal agricultural community.

### 3.3 Masculinities perspective in the session on personal reflections on gender

**Figure 2:**
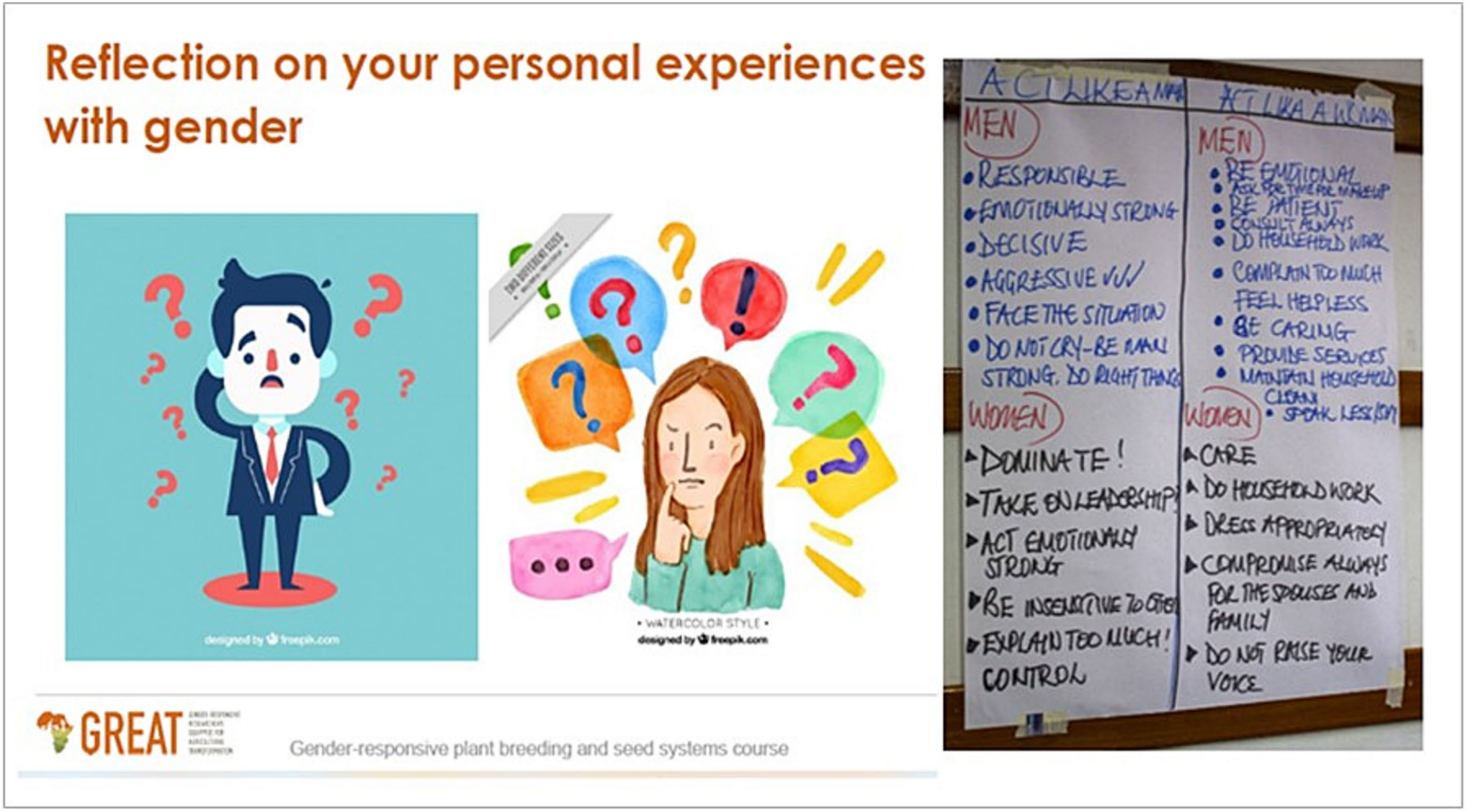
Flip chart group output, case study 1, September 2022, India.

Masculinities perspective was also introduced in a session on “Personal reflections on Gender.” Prior to the newly integrated perspective, the session required participants to reflect on their personal lives, their workplaces and the professional agricultural disciplines they came from and trace how women’s experiences of marginalization manifested. With the introduction of masculinities approach, the training session broadened the focus on critical reflections aimed at fostering discovery and realization of how gender manifests at personal, workplace, and discipline levels, including through participatory exercises on how men’s experiences impact women empowerment outcomes. One exercise instructed thus: In separate groups of men and women, discuss and write down what comes to your mind when someone says: (a) act like a man! (b) act like a woman! These reflections are illustrated in Figure 2.

The participatory exercises on who an ideal man is in a given social-cultural context were found to be particularly powerful and revealing. A female biophysical Indian trainer marveled thus:

*“How did we manage to get Indian men to open up and discuss experiences of vulnerabilities they go through? This is not an Indian practice, for men to speak out the way they did during the session on masculinities!”*

### 3.4 Independent session on masculinities and agriculture

The independent session on men in Agriculture was introduced in 2019, initially allocated 25 min, in relation to others that carried an hour plus of training time. Over time, the session on masculinities in agriculture gained traction, with participants requesting to learn more about men’s preponderant power in agriculture value chains. In the selected case studies, the session was allocated 1 h to enable it include diverse interactive exercises.

The concept of masculinities was always linked to the theme of training (e.g., plant breeding and seed systems for Case 1 and agronomy for Case 2), and the cultures of the regions hosting the training (South Asia and Sub-Saharan Africa respectively). In terms of delivery, these sessions were equally participatory, culturally rooted, and gave freedom to the participants to learn from each other and their environments.

**Figure 3:**
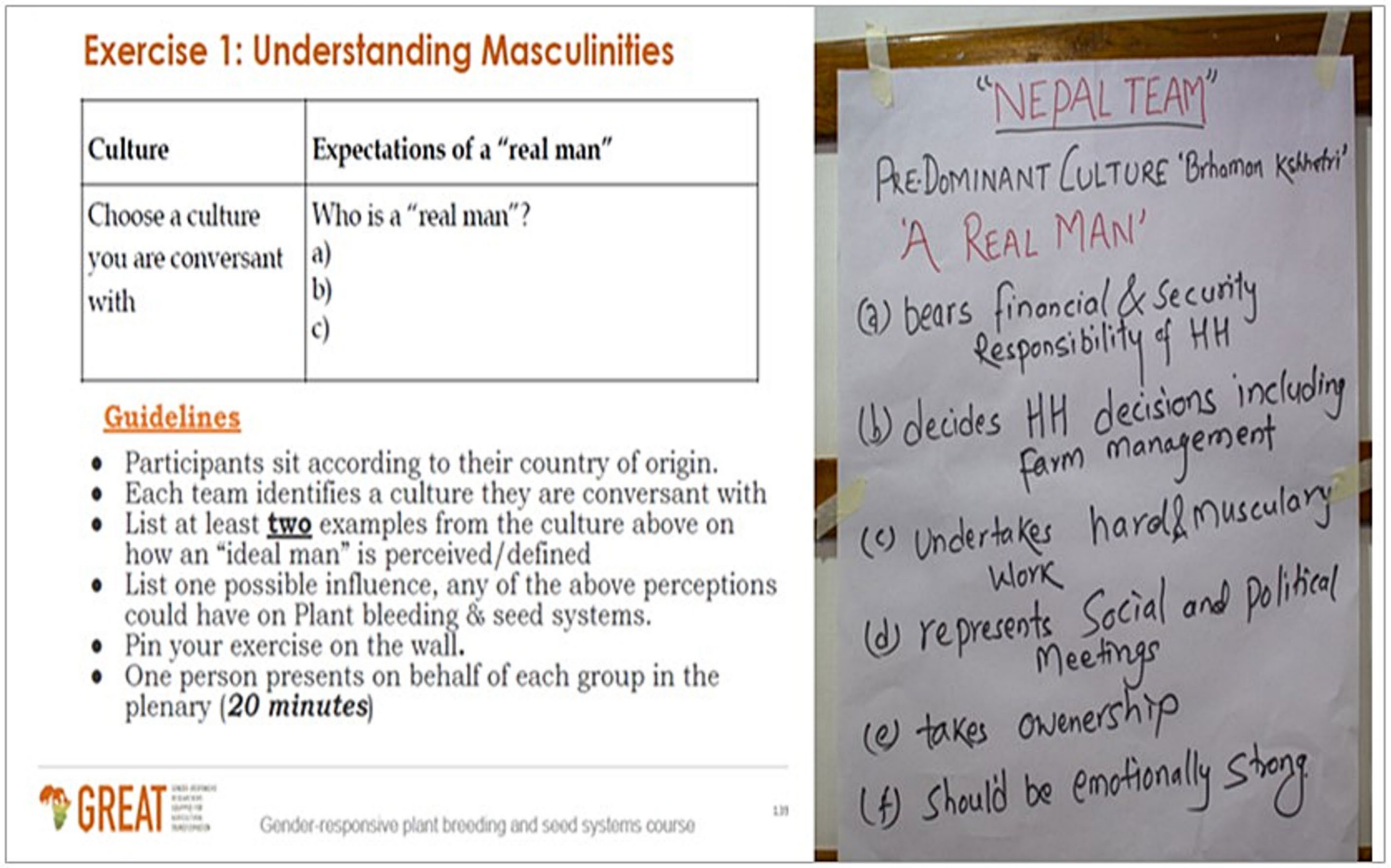
Participants’ group output, case study 1, September 2022, India.

**Figure 4:**
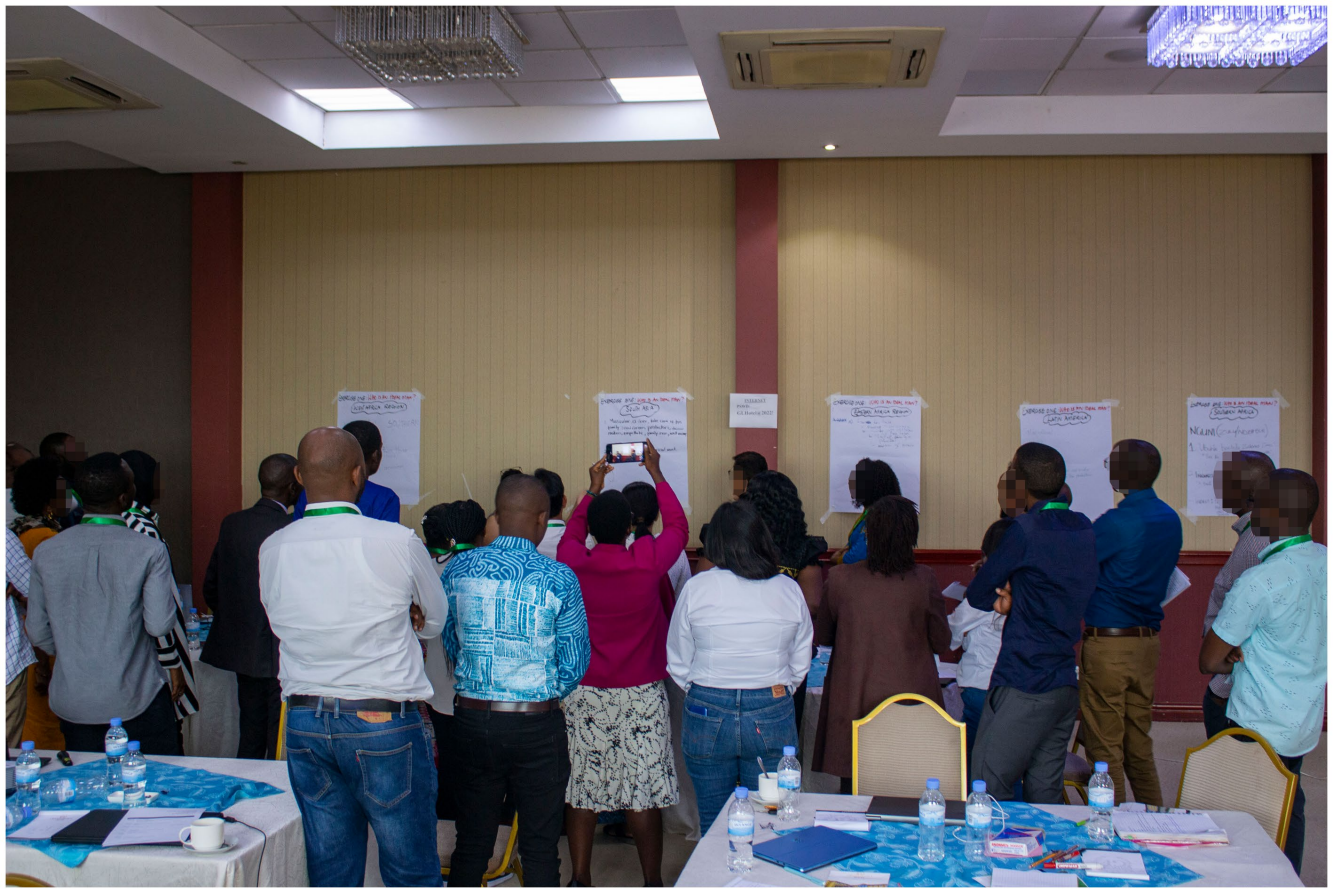
Participants listening to colleagues’ presentation.

The session aimed to enhance participants’ understanding of the concept and its relevance in agricultural research and development practice drawing on social theories on masculinities for example hegemonic masculinities Connell (2005), and its application in agriculture (Brandth, 1995; Saugeres, 2002; Cole et al., 2015). The session covered theories on masculinities to enable participants to appreciate the growth of these debates and the implications these theoretical resources have for agricultural research. The figure below illustrates practical exercises that participants go through to define and appreciate social constructions of masculinities (see Figures 3, 4).

One of the participatory assignments in the training session includes requesting participants to reflect on cultures they are conversant with, describe cultural constructions of ‘ideal’ manhood, trace the dominant images of men that emerge, and assess the impact of these on agricultural practices. Group discussions often gave participants an opportunity to look at how different cultures constituted men’s roles and expectations differently but also how these masculine identities vary within societies, change from time to time as well as how they are constructed in relation to femininities.

Despite the diversity in geographical and social-cultural zones, participants listed closely similar social expectations associated with being a man. These expectations exhibited patterns associating men with symbolic power, control, and influential decision-making. For example, among the Nguni of Zimbabwe, masculinities are constructed through symbolic language that points to power. The Nguni argue that “the beauty of a man is in his cows,” which points to control over wealth and possession as a key attribute of being a man. They also have the saying that “a bull is worthy of its scars,” which speaks to the association of men with conquest, aggression, and dominance. These widely celebrated proverbs and sayings “put men under pressure to amass property, conquer, and dominate, and that men who do not exhibit these behaviors feel culturally ‘disempowered’ and may become bullies in an attempt to reclaim their perceived loss of masculine power” (Male participant, Case 2). Participants in Case 2 then argued for the need to always interrogate the often taken-for-granted experiences of men as part of gender analysis because of this perspective’s potential to pave the way toward understanding persistent gender inequalities and possibilities of transforming these.

This training session also focused on historicizing the growth in the focus on men in gender analysis (Sweetman, 1997; Cornwall, 2000), the work on women’s empowerment (Sraboni et al., 2014; Akter et al., 2017; Bonatti et al., 2019), and gender transformation (FAO, IFAD, and WFP, 2020). Further participatory exercises focus on understanding the value addition of focusing on men and masculinities in agriculture. In these exercises, participants recalled how understanding masculinities enables clear picture of who does what in agriculture (gender division of labor), tracing of decision-making patterns, e.g., who to consult, and involve while introducing agricultural innovations, understanding culturally sanctioned men’s expectations and behaviors that hinder and/or enable agricultural productivity. It has also been argued that excluding men in agricultural innovations, research, and training is often detrimental to positive change, e.g., through provoking male hostility such as men withdrawing from agricultural labor (Cleaver, 2002; Mwiine, 2022).

The independent session on masculinities was appreciated for being an eye-opener towards understanding the importance of integrating men’s perspectives in gender and agronomy projects (Case 2). In one of the Key informant interviews, a male agronomist participant expressed these sentiments as follows.

*The issue of masculinity is something that I had never heard of and it is still fresh in my memory. These things happen in communities, but we do not talk about them…It was my first gender training and masculinity caught my attention in the extreme, that’s quite interesting. When you look back in the communities we work with, men will always want to take their position and we should know how such matters affect our work” (KII, Man, agronomist).*

These reflections point to the participant’s appreciation of a new conceptual debate in gender relations, an expansion of gender analysis and most importantly the fact that the training revealed often taken-for-granted everyday experiences of masculine relations that influence agricultural practices. In Case 1, participants applauded the course for including pertinent topics not usually part of gender training courses, with particular reference to the session on masculinities as unanticipated.

### 3.5 Integrating masculinities lens in gender analysis frameworks

Prior GREAT Training on gender analysis drew heavily on tracing experiences of discrimination and disadvantage amongst women farmers. This perspective was expanded through integrating a masculinities perspective in gender and agriculture analysis. The framework builds on the Social Relations Model by Kabeer and Subrahmanian (1996). The analytical frame, which has been widely used to analyze women’s position in social institutions focuses on 5 key domains, i.e., rules, activities, resources, people and power. While this framework has the potential to analyze women and men’s relationships in social institutions, it has been commonly used to analyze women’s relationships. In the session, we suggest key areas in which men’s relationships in organizations could be analyzed along parameters of production and income, resources, leadership, time, and cost/constraints. Questions under each of the key parameters that guided participants are listed below:

**Table:** 

**How to analyze masculine norms in agriculture** **Production’ and ‘income’**: Who does what, who gets what, and who can claim what? Who decides, and whose interests are served? **Resources**: Which resources do we have? Who accesses/controls? **Leadership**: Which groups do men belong to? Which ones are they absent from and why? How do men exercise power and leadership in groups? **Time**: How much time do men allocate to productive and domestic tasks and leisure activities? **Constraints/Cost**: What constraints do men have in our communities? How do masculine norms constrain men’s participation in agriculture practices?

The training session on men and masculinities also included participatory reflections on gender transformative approaches. Notions of men and masculinities in the session on gender transformative approaches involved guided discussions on gender norms, “the unwritten, informal social rules that determine socially acceptable behavior for men and women” (Hillenbrand and Miruka, 2019). The key message here is that gender transformation requires questioning feminine as well as masculine norms. The session also reflected on explicitly engaging with men and boys to address the concepts of masculinity (FAO, IFAD, and WFP, 2020) as one of the key strategies to ensure gender transformation.

### 3.6 From training sessions to conducting field research using the masculinities approach

Over a 20-month period from May 2021 to December 2022, early career fellows who had completed the theoretical training sessions were recruited into a research cluster on *Women empowerment, Masculinities, and Social norms.* The approach involved experiential research activities such as hands-on mentorship in critical reading, writing, and conducting research. Fellows and trainers collectively participated in the systematic research process from conceptualization, and implementation to publication of research results. This provided an opportunity to learn together, innovatively developing frameworks on how to search for, sieve, and arrive at the literature on agriculture and masculinities, develop research gaps, develop research methods, collect and analyze data on men and women’s experiences in agricultural communities.

## 4 Discussion of findings

When GREAT introduced training programmes for agricultural researchers and practitioners through a feminist lens, its motive was to offer a comprehensive, trainee-centered, critical, interdisciplinary, and practical gender training course. The training course would provide a space for reflection on internalized gender beliefs, biases, and identities and foster gender transformation (Mangheni et al., 2019). While the training course set out to critique unequal gender power relations in agricultural research, it did not originally deliberately set out to interrogate notions of masculinities and their implication on the journey of transformation.

The focus on masculinities perspective and later (after several cohorts of course offerings) its intentional integration in the GREAT model emerged inductively from Trainers’ and participants’ critical reflections on how individuals, workplaces, and research processes become gendered. The critical reflections on personal experiences gave participants an opportunity to discover beliefs and perceptions about being a man and a woman, the biases and inequalities that come from these, and how workplaces, research processes, and other social institutions acquire and normalize these gendered beliefs and practices.

The inclusion of men as a category of analysis widened participants’ scope of reflection to include how both women and men become gendered. The exercises highlighted how men (like women) are equally products of social learning processes (Mwiine and Katushabe, 2024; Kikooma et al., 2023). Participatory and reflective methods of learning processes of this nature empower the learners by encouraging participation, self-discovery, and creative learning (Shrewsbury, 1987).

Feminist pedagogies indicate that one goal of the liberatory classroom (a concept central to feminist pedagogy) is that members learn to respect each other’s differences rather than fear them (Mangheni et al., 2021). Consequently, interrogating men’s experiences respectfully gained presence in GREAT training sessions, from a mere focus on the definition of masculinities and femininities to the introduction of a fully-fledged session on masculinities and agriculture.

From concepts on masculinities to gender analysis approaches that unpack men’s experiences in agricultural communities, the masculinities perspective provided an opportunity to understand how men’s preponderant power in agricultural communities comes about, raising participants’ consciousness on how men’s experiences are implicated in gender power relations. It also acts as an entry point in understanding the roots of male resistance to gender change. To realize that men’s behaviors are social, can and indeed do change, is a great step towards gender transformation (Shefer et al., 2022; Mwiine and Katushabe, 2024).

### 4.1 Socio-cultural differences and convergencies between the two case studies

This study purposefully focused on two training courses as case studies to understand participants’ appreciation of the focus on masculinities. The courses occurred in social-culturally and disciplinary diverse contexts of gender, class, ethnicity, caste, religion, age, geographical location, covering different themes in agricultural research. For instance case one’s focus on the Asian sub-continent (India, Bangladesh, Nepal) provided an opportunity to reflect on the constitution of masculinities within historical and rigid institutions of religion, caste and creative programmes on women’s empowerment. While religion, culture, castes might dictate rigid forms of male behavior, feminist pedagogy methods availed a space where these forms of masculinity were reflected upon by women and men participants and trainers, enabling critical level of consciousness abount masculine domination and vulnerabilities at the same time in Asian agricultural communities. Indeed, a female biophysical Indian trainer marveled “How did we manage to get Indian men to open up and discuss experiences of vulnerabilities they go through? This is not an Indian practice …!” The marvel at the men’s readiness to open up and discuss issues that would otherwise be seen as unmanly (men’s expression of emotions and admission of vulnerability) (UNESCO New Delhi, 2021) points to critical consciousness (triggered by men and women’s collegial reflection on gender relations) as key to gender transformation (FAO, IFAD, and WFP, 2020).

The second case study which featured participants mostly from Sub-Sahara Africa (Rwanda, Ghana, Nigeria, Benin, Ethiopia, Malawi, Cambodia, India), showed how, despite cultural diversity, participants noted closely similar masculine identities in their agricultural communities. Drawing from their cultural and linguistic repertiores, they highlighted normalisation of male domination in resource ownership, decision making and mostly men’s reluctance to change for fear of being seen as not men-enough.

Despite rigid social institutions that police and reinforce men’s dominant behavior, critical reflections through feminist pedagogy principles indicated how masculinities can and, and do change towards gender equality. Similar argument of diversity of male behavior showing instances where men exhibit progressive and gender equitable behaviors is advanced by Su (2024). While examining experiences of men in Vietnam, Su shows how, amidst socioeconomic transitions, men in low-paid jobs aspired for the notion of ‘traditional’ male breadwinner and a caretaker wife while the men in salary earning contexts prefered a dual-income family, viewing women workers as progressive.

The study demonstrated how consciousness-raising, as a first step towards transforming unequal gender relations, requires engaging women and men together, to subvert traditional norms and practices that constrain equitable relations. While men’s change towards progressive behavior often attracts punishments (e.g., how did we get Indian men to speak about their vulnerabilities? This is not Indian Practice! Or Sub-Sahara men’s fear to change), it is through such subversive acts (going against the norm) that create possibilities of gender transformation (Butler, 1988; Mwiine, 2020). Butler argues that “… the possibilities of gender transformation are to be found in the arbitrary relation between such acts, in the possibility of a different sort of repeating, in the breaking or subversive repetition of that style” (1988; 520).

## 5 Conclusion

The GREAT training model demonstrates how to introduce masculinities in a feminist gender training course. First, the concept of masculinities was defined alongside other gender concepts such as femininities, gender relations, and gender equality. Second, an independent session offers more in-depth coverage of masculinities theory and application in agriculture. Third, masculinities was covered as part of gender norms and gender transformation, and finally, through experiential learning during field research.

The use of a feminist pedagogical approach which affords participants opportunities for critical reflection on personal, organizational, and cultural experiences with gender revealed how masculinities are implicated in gender power relations. Importantly, the process of teaching and learning demonstrably shifted the debate on gender training, moving beyond the conventional focus on women and men as gender categories. Intentional examination of men’s experiences within gender and agriculture training contributes towards the body of knowledge that acknowledges masculinities as complementary rather than being opposed to feminism. Feminist pedagogical practices also offer insights into how gender training can integrate a masculinities perspective to move beyond divisive and narrow gender polarities towards addressing masculine norms that often hinder the attainment of gender transformation.

### 5.1 Limitations of the study

The paper is based on an in-depth analysis of a unique case study. It relied heavily on participants’ self-reported data and trainers’ reflections, which could have been influenced by various contextual factors. Generalizability to other gender training courses in different contexts would require contextual adaptation. Although the study findings have implications for transformative gender training in agriculture and beyond, the GREAT model itself and the masculinities pedagogy require further testing in other contexts.

## Data Availability

The original contributions presented in the study are included in the article/supplementary material, further inquiries can be directed to the corresponding author.
